# Impact of prior intravenous thrombolysis on first-line thrombectomy strategy. A secondary analysis of the VECTOR trial

**DOI:** 10.1093/esj/aakag087

**Published:** 2026-07-24

**Authors:** Samuel J Mouyal, Benjamin Maïer, Julien Labreuche, Jean-Philippe Desilles, Mikael Mazighi, Romain Bourcier, Hocine Redjem

**Affiliations:** Interventional Neuroradiology Department, Rothschild Foundation Hospital, Paris, France; Interventional Neuroradiology Department, Rothschild Foundation Hospital, Paris, France; Neurology Department, Hôpital Paris Saint-Joseph, Paris, France; FHU NeuroVasc, INSERM UMRS 1144, Université Paris Cité, Paris, France; Stroke-Link F-CRIN Research Network, Lille, France; EA 2694-Santé Publique: épidémiologie et qualité des soins, University of Lille, CHU Lille, Lille, France; Interventional Neuroradiology Department, Rothschild Foundation Hospital, Paris, France; FHU NeuroVasc, INSERM UMRS 1144, Université Paris Cité, Paris, France; Stroke-Link F-CRIN Research Network, Lille, France; Interventional Neuroradiology Department, Rothschild Foundation Hospital, Paris, France; FHU NeuroVasc, INSERM UMRS 1144, Université Paris Cité, Paris, France; Stroke-Link F-CRIN Research Network, Lille, France; Neurology Department, Hôpital Lariboisière, APHP, Institut Universitaire de France, Paris, France; Interventional Neuroradiology Department, CHU Nantes, Nantes, France; Interventional Neuroradiology Department, Rothschild Foundation Hospital, Paris, France

**Keywords:** acute ischaemic stroke, LVO, mechanical thrombectomy

## Abstract

**Introduction:**

Whether prior intravenous thrombolysis (IVT) modifies the relative efficacy of first-line thrombectomy strategies remains uncertain, and no randomised trial has specifically addressed this question. Leveraging the randomised design of the VECTOR trial, a multicentre trial comparing first-line contact aspiration (CA) vs CA plus stent retriever (SR + CA) in patients with anterior LVOs and a positive susceptibility vessel sign, we assessed whether IVT status modified the association between thrombectomy strategy and reperfusion outcomes.

**Patients and methods:**

We performed a secondary analysis of the VECTOR trial, in which patients were stratified according to prior IVT use. The primary outcome was near-complete or complete reperfusion (expanded thrombolysis in cerebral infarction [eTICI] 2c-3) after ≤ 3 passes with the assigned first-line technique and before rescue therapy. Secondary outcomes included first-pass reperfusion, complete reperfusion after ≤ 3 passes, final reperfusion at the end of the procedure and functional outcomes at 90 days.

**Results:**

Between 26 November 2019 and 14 February 2022, 521 patients were included in VECTOR. Of these, 288 (55%) received IVT before thrombectomy. The effect of first-line strategy on the primary outcome did not differ according to IVT status (*P* for heterogeneity = .28). Among IVT-treated patients, eTICI 2c-3 after ≤ 3 passes occurred in 88/144 patients (61.1%) assigned to SR + CA vs 74/144 (51.3%) assigned to CA (aOR, 1.50 [95% CI, 0.91–2.45]). Among patients without IVT, corresponding rates were 64/119 (53.9%) vs 61/114 (53.6%) (aOR, 1.00 [95% CI, 0.58–1.72]). Interaction tests were non-significant for all other angiographic, clinical and safety outcomes except final complete reperfusion, which favoured CA in patients without IVT (*P* for heterogeneity = .019).

**Discussion:**

Prior IVT did not significantly modify the effect of first-line strategy on early reperfusion, and both CA and SR + CA remained valid approaches irrespective of IVT status. The isolated interaction for final complete reperfusion, favouring CA in patients without IVT, was exploratory and may reflect chance. A hypothesis-generating mechanism is plausible in this SVS-positive, red blood cell–rich phenotype: CA applied to an intact clot may favour en-bloc retrieval, an advantage attenuated once alteplase softens the thrombus. These results, obtained almost exclusively under alteplase and in an MRI-selected population with mandatory balloon guide catheter use, may not extend to tenecteplase or CT-selected patients.

**Conclusions:**

In this secondary analysis of the VECTOR trial, prior IVT did not significantly modify the association between first-line thrombectomy strategy and successful reperfusion. The observed angiographic differences remain exploratory and warrant further investigation.

## Graphical Abstract

**Graphical Abstract:**
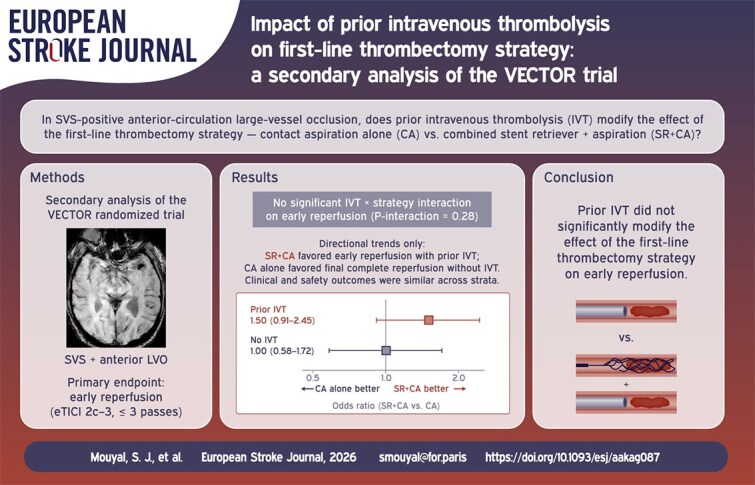

## Introduction

Mechanical thrombectomy (MT) is the standard of care for acute ischaemic stroke due to anterior circulation LVO, supported by multiple randomised trials and pooled individual-patient meta-analyses showing substantial clinical benefit when timely reperfusion is achieved.^1–5^ In contemporary practice, first-line MT is most commonly performed using direct contact aspiration (CA) or stent-retriever-based strategies (SR), including combined techniques pairing aspiration with a stent retriever (SR + CA). Randomised trials comparing these approaches in unselected LVO populations have reported broadly similar overall angiographic and clinical outcomes, yet important heterogeneity persists and early, near-complete reperfusion is not consistently achieved.^6^^,^^7^ Identifying factors that modify the relative performance of first-line strategies therefore remains a key clinical need.

One potential modifier is prior use of intravenous thrombolysis (IVT).^8^ Indeed, IVT may alter thrombus architecture and device-clot interaction, thereby influencing the efficacy of specific MT techniques. Recent post-hoc analyses have suggested that the impact of IVT may differ according to the first-line endovascular approach, with prior administration of alteplase potentially reducing the efficacy of first-line CA strategies while having little or no effect on SR-based approaches.^9^^,^^10^ By contrast, the impact of IVT on the relative efficacy of CA alone vs combined SR + CA approaches remains largely unexplored, with available data mostly being observational. This question is clinically relevant for 2 main reasons. First, CA alone and combined SR + CA approaches have progressively become the 2 most widely used first-line MT strategies in anterior circulation LVO. Second, several recent randomised trials have reinforced the role of bridging IVT in this population, highlighting the need to better understand whether IVT status modifies the comparative performance of these endovascular strategies.^11^

The VECTOR trial provides a unique opportunity to address this question. In this randomised trial, patients with anterior LVO and a positive susceptibility vessel sign (SVS) were assigned to first-line CA alone or SR + CA.^12^ We, therefore, performed a secondary analysis of the VECTOR dataset to investigate the interaction between IVT status and first-line endovascular strategy.

## Patients and methods

### Study design and data source

This study is a secondary analysis of the VECTOR trial, which was a multicentre, prospective, randomised, open-label trial with blinded outcome evaluation conducted in 22 comprehensive stroke centres in France.^12^ The present analyses used the original trial data and procedures as collected in VECTOR; the full trial design, conduct and procedural details are reported in the primary VECTOR publication.^12^ Although the VECTOR trial is a randomised controlled study, the present analysis is a secondary, non-prespecified subgroup analysis and should therefore be considered observational in nature. Accordingly, this study was conducted and reported in accordance with the Strengthening the Reporting of Observational Studies in Epidemiology (STROBE) guidelines.^13^

### Ethics and consent

The VECTOR trial was conducted in accordance with the Declaration of Helsinki and Good Clinical Practice, with ethics approval at each participating centre and emergency consent procedures compliant with French regulations.

### Participants

VECTOR enrolled adults (≥18 years) with acute ischaemic stroke due to proximal anterior circulation LVO involving the internal carotid artery terminus or the M1 or proximal M2 segment of the MCA, with a clear SVS on baseline MRI (T2^*^ gradient-echo, susceptibility-weighted imaging or related susceptibility sequences) and arterial puncture for MT within 24 h of symptom onset. Intravenous thrombolysis before MT was permitted according to local practice and European guidelines.^14^ As the trial was conducted during a period of tenecteplase shortage, intravenous alteplase was the main thrombolytic agent administered.

Key exclusion criteria in VECTOR included posterior circulation occlusion, distal M2 occlusion, angiographic carotid dissection or tandem cervical carotid occlusion/stenosis requiring specific treatment, known or suspected chronic intracranial occlusion in the symptomatic territory, severe contrast allergy, pre-stroke mRS score >1, severe comorbidities limiting recovery or follow-up, pregnancy and breastfeeding.

### Randomisation and masking

Patients were randomised (1:1) to first-line CA alone or a combined SR + CA technique (Embotrap, Cerenovus), using central web-based allocation with a minimisation procedure balancing centre and prespecified prognostic variables including prior IVT use. Angiographic outcomes, including expanded thrombolysis in cerebral infarction (eTICI) gradings, were adjudicated by an independent imaging core laboratory blinded to treatment allocation and clinical status. Clinical assessments at 24 h and 90 days were also performed by personnel blinded to group allocation in the parent trial.

### Procedures

Baseline imaging consisted of MRI in all patients, performed per each centre’s routine to exclude intracranial haemorrhage and to document anterior circulation occlusion; SVS was defined as a hypointense signal at the occlusion site exceeding the diameter of the contralateral vessel. To reflect clinical practice, susceptibility imaging parameters were not standardised across centres, but enrolling physicians completed a prespecified SVS certification process before participating.

Mechanical thrombectomy procedures were performed under general anaesthesia or conscious sedation. Of note, the use of a balloon guide catheter was mandated for both strategies in the study protocol. The combined strategy was performed using an SR + CA approach with an *en bloc* retrieval of the aspiration catheter and stent retriever under flow arrest and aspiration through the balloon guide catheter (ie, BADASS technique^15^). In the CA arm, a large-bore aspiration catheter was advanced to the proximal face of the thrombus, aspiration was applied for a prespecified duration and the catheter was withdrawn under proximal flow arrest. The allocated first-line technique was repeated until near-complete or complete reperfusion was achieved; at least 3 passes with the assigned technique were required before switching to an alternative approach (rescue therapy).

### Outcomes

The primary outcome was near-complete or complete reperfusion, defined as an eTICI 2c-3 achieved after 3 or fewer passes with the assigned first-line technique and before the use of rescue therapy, on the immediate post-treatment angiogram as adjudicated by a blinded core laboratory. This endpoint was deliberately identical to the primary outcome of the parent VECTOR trial to ensure methodological consistency and interpretability. In cases of crossover, the eTICI grade obtained before switching technique was considered, consistent with the endpoint definition of the parent trial.

Secondary angiographic endpoints included the proportion of first-pass effect with the allocated first-line technique, using 2 definitions: near-complete or complete reperfusion (eTICI 2c-3) and complete reperfusion (eTICI 3). Other secondary endpoints included the proportion of complete reperfusion (eTICI 3) achieved after 3 or fewer passes of the assigned first-line strategy, the proportion of patients with eTICI 2c-3 and eTICI 3 at the end of MT (ie, using the first-line strategy or rescue therapy).

Secondary clinical endpoints included the change in the NIHSS score between baseline and 24 h and 90-day functional outcome assessed with the mRS. The mRS was analysed both ordinally (with scores 5 and 6 combined) as well as dichotomised into favourable outcome (mRS 0–2 vs 3–6) and excellent outcome (mRS 0–1 vs 2–6).

Safety outcomes consisted of procedure-related adverse events, ICH at 24 h (any ICH, parenchymal haematoma and symptomatic ICH defined according to the ECASS II definition^16^), and all-cause mortality at 90 days.

### Statistical analysis

All analyses were done in the full analysis set including all randomised patients, regardless of their eligibility and any protocol deviations, according to the first-line MT strategy to which they were assigned at randomisation (combined technique vs contact aspiration); only patients who dropped out before catheter angiography (*n* = 2) or consent withdrawal (*n* = 3) were excluded.^12^ Baseline characteristics were described according to the allocated first-line MT strategy and prior use or not of intravenous thrombolysis. To assess the potential imbalance in baseline characteristics between the intervention group of interest (first-line combined technique vs contact aspiration alone) in IVT-treated patients and non-IVT-treated patients separately, we calculated the standardised difference; an absolute standardised difference >20% was considered to be a meaningful imbalance.

In IVT-treated patients and non-IVT-treated patients separately, as main analysis, we estimated the effect sizes of the first-line combined technique over contact aspiration alone strategy on primary endpoint and secondary outcomes by using multivariable regression models controlling for the randomisation stratification variables, by including age (80≤ vs >80), prior use of IVT, occlusion site (isolated MCA vs MCA/internal carotid artery) and general anaesthesia as fixed effects and centre as random effect. The primary outcome and all second binary outcomes were analysed using a mixed logistic regression model and adjusted odds ratio (aOR) were derived as effect size. The secondary outcomes were analysed using a mixed ordinal logistic regression model for ordinal outcomes [distribution of the 90-day mRS score after combining scores of mRS 5 and 6 together], a constrained longitudinal data analysis (cLDA) model for change in NIHSS from admission to 24 h (to adjust for baseline value in addition to the randomisation stratification variables) and using a marginal Fine and Grey model for arterial puncture to eTICI 2c or better reperfusion time (considering eTICI 2c or better as event of interest and the end endovascular procedure without eTICI 2c as competing event). Effect sizes were, respectively, expressed in term of adjusted common odds ratio for 1-point improvement in mRS score, adjusted mean difference in NIHSS change, and adjusted sub-hazard ratio for the time from arterial puncture to eTICI2c or better reperfusion. Effect sizes for combined SR and CA first-line strategy vs first-line CA alone strategy were reported in IVT-treated and non-IVT-treated patients by using a forest plot and were compared using chi-square test for heterogeneity. We also estimated effect sizes after additional adjustment on baseline characteristics showing meaningful imbalance between treatment groups within the IVT and non-IVT subgroups (standardised difference >20%), except for glucose level and collateral status because of the substantial proportion of missing data. Primary analyses were conducted after handling missing outcomes values (ranged from 2.1% for 24-h intracranial haemorrhage to 9.6% for 24-h NIHSS score) by multiple imputations under the assumption of missing at random, using fully conditional specification based on treatment group and baseline characteristics (*m* = 20 imputations).^17^ In each imputed dataset, we estimated the effect sizes, which were later combined using Rubin’s rules. Analyses conducted before handling missing outcomes values are reported as sensitivity analysis (see Figure S1).

Data were analysed using the SAS software package, version 9.4 (SAS Institute, Cary, NC). Statistical tests were 2-sided, and a *P*-value < .05 was considered statistically significant. Due to the potential for type I error from multiple comparisons, findings should be considered exploratory.

## Results

Between 26 November 2019 and 14 February 2022, 526 patients were enrolled in the VECTOR trial; 265 patients were randomly assigned to undergo SR + CA as first-line strategy and 261 to undergo CA alone. After excluding 5 patients who dropped out before catheter angiography (*n* = 2) or consent withdrawal (*n* = 3), 521 patients were analysed. Of them, 288 (55%) received IVT before MT. Among IVT-treated patients, 144 were allocated to the SR + CA strategy and 144 to CA alone. In patients without IVT, 119 were allocated to the SR + CA strategy and 114 to CA alone. Baseline characteristics according to allocated first-line MT strategy and IVT use are presented in Table 1. Baseline characteristics were well balanced across treatment strategies within each IVT stratum, with only moderate imbalances noted. In the IVT subgroup, M2 occlusions were more frequent in the SR + CA group compared with the CA alone group (29/144 [20.1%] vs 16/144 [11.1%]). Conversely, general anaesthesia was used more often in the CA alone group (37/144 [25.7%] vs 24/142 [16.9%]). In patients without IVT, the onset-to-groin puncture time was longer in the SR + CA group (median 277 vs 242 min).

**Table 1:** Patients’ characteristics at baseline, according to the allocated first-line thrombectomy strategy and the use of intravenous thrombolysis prior thrombectomy.

Characteristics	No prior IVT: SR + CA first-line strategy (*n* = 119)	No prior IVT: CA alone first-line strategy (*n* = 114)	Standardised difference, %	Prior IVT: SR + CA first-line strategy (*n* = 144)	Prior IVT: CA alone first-line strategy (*n* = 144)	Standardised difference, %
**Age (years)**	76 (67–85)	75 (64–83)	−10.9	74 (63–83)	74 (64–83)	0.3
**Gender**						
**Men**	53/119 (44.5)	47/114 (41.2)	−6.7	67/144 (46.5)	70/144 (48.6)	4.2
**Women**	66/119 (55.5)	67/114 (58.8)	−6.7	77/144 (53.5)	74/144 (51.4)	
**Body mass index (kg/m^2^)**	25.0 (22.8–29.4) [*n* = 95]	25.4 (22.8–29.1) [*n* = 95]	1.6	26.2 (23.8–28.4) [*n* = 108]	25.5 (23.6–29.0) [*n* = 110]	−7.5
**Medical history**						
**Hypertension**	71/118 (60.2)	65/113 (57.5)	−5.4	77/140 (55.0)	82/143 (57.3)	4.7
**Diabetes**	13/117 (11.1)	16/114 (14.0)	8.8	20/138 (14.5)	21/142 (14.8)	0.8
**Hypercholesterolaemia**	39/115 (33.9)	38/111 (34.2)	0.7	39/135 (28.9)	35/139 (25.2)	−8.4
**Current smoking**	17/90 (18.9)	15/96 (15.6)	17.8	20/100 (20.0)	21/107 (19.6)	23.9
**Coronary artery disease**	19/117 (16.2)	18/113 (15.9)	−0.8	19/137 (13.9)	13/139 (9.4)	−14.1
**Previous stroke or TIA**	18/116 (15.5)	17/113 (15.0)	−1.3	21/139 (15.1)	11/142 (7.7)	−23.3
**Previous antithrombotics**	53/117 (45.3)	48/113 (42.5)	−5.7	45/138 (32.6)	34/142 (23.9)	−19.3
**Antiplatelet**	28/117 (23.9)	19/113 (16.8)	−17.8	35/138 (25.4)	24/142 (16.9)	−20.8
**Anticoagulant**	27/117 (23.1)	30/113 (26.5)	8	10/138 (7.2)	10/142 (7.0)	−0.8
**Current stroke event**						
**Mode of admission**						
**Drip-and-ship**	54/119 (45.4)	53/111 (47.7)	−4.8	55/143 (38.5)	55/143 (38.5)	0
**Mothership**	65/119 (54.6)	58/111 (52.3)		88/143 (61.5)	88/143 (61.5)	
**Admission systolic BP (mmHg)**	149 (25) [*n* = 116]	145 (25) [*n* = 106]	−13.8	147 (26) [*n* = 135]	145 (28) [*n* = 136]	−7.3
**Admission diastolic BP (mmHg)**	83 (16) [*n* = 115]	81 (17) [*n* = 107]	−15.4	84 (16) [*n* = 134]	82 (17) [*n* = 136]	−13.6
**Admission NIHSS score**	17 (13–21) [*n* = 117]	17 (14–21)	0.5	16 (11–21) [*n* = 143]	16 (13–20)	1.6
**Admission glucose (g/L)**	1.3 (1.1–1.5) [*n* = 102]	1.2 (1.1–1.4) [*n* = 89]	−13.5	1.2 (1.0–1.4) [*n* = 116]	1.3 (1.1–1.5) [*n* = 115]	23.1
**Pre-stroke mRS**						
**0**	94/117 (80.3)	88/114 (77.2)	25.5	111/140 (79.3)	124/143 (86.7)	25.4
**1**	20/117 (17.1)	19/114 (16.7)		20/140 (14.3)	15/143 (10.5)	
**2**	1/117 (0.9)	2/114 (1.8)		3/140 (2.1)	1/143 (0.7)	
**3**	1/117 (0.9)	4/114 (3.5)		6/140 (4.3)	1/143 (0.7)	
**4**	1/117 (0.9)	1/114 (0.9)		0/140 (0.0)	2/143 (1.4)	
**Admission ASPECTS**	7 (5–8) [*n* = 114]	7 (5–8) [*n* = 109]	−5.9	8 (6–9) [*n* = 139]	8 (6–9) [*n* = 134]	3.5
**Site of occlusion**						
**M1-MCA**	80/119 (67.2)	78/114 (68.4)	3.9	90/144 (62.5)	102/144 (70.8)	28.9
**M2-MCA**	10/119 (8.4)	8/114 (7.0)		29/144 (20.1)	16/144 (11.1)	
**Intracranial ICA**	25/119 (21.0)	24/114 (21.1)		24/144 (16.7)	25/144 (17.4)	
**Extracranial ICA**	4/119 (3.4)	4/114 (3.5)		1/144 (0.7)	1/144 (0.7)	
**Clot burden score**	7 (6–8) [*n* = 113]	7 (6–8) [*n* = 105]	4.4	8 (6–8) [*n* = 139]	7 (6–8) [*n* = 133]	−6.2
**Collaterals**						
**Grade 0**	9/99 (9.1)	7/94 (7.4)	14.7	11/116 (9.5)	15/124 (12.1)	23.1
**Grade 1**	17/99 (17.2)	16/94 (17.0)		24/116 (20.7)	19/124 (15.3)	
**Grade 2**	26/99 (26.3)	30/94 (31.9)		32/116 (27.6)	28/124 (22.6)	
**Grade 3**	42/99 (42.4)	36/94 (38.3)		39/116 (33.6)	49/124 (39.5)	
**Grade 4**	5/99 (5.1)	5/94 (5.3)		10/116 (8.6)	13/124 (10.5)	
**Suspected stroke cause**						
**Large artery atherosclerosis**	9/93 (9.7)	9/88 (10.2)	13.7	14/105 (13.3)	10/112 (8.9)	22.4
**Cardioembolism**	60/93 (64.5)	52/88 (59.1)		56/105 (53.3)	67/112 (59.8)	
**Other determined cause**	5/93 (5.4)	5/88 (5.7)		6/105 (5.7)	10/112 (8.9)	
**Undetermined or multiple cause**	19/93 (20.4)	22/88 (25.0)		29/105 (27.6)	25/112 (22.3)	
**Directly admitted to a comprehensive stroke centre**	54/119 (45.4)	53/111 (47.7)	−4.8	55/143 (38.5)	55/143 (38.5)	0
**Anaesthesia**						
**General**	31/117 (26.5)	28/112 (25.0)	14.8	24/142 (16.9)	37/144 (25.7)	23.1
**Local**	83/117 (70.9)	83/112 (74.1)		115/142 (81.0)	105/144 (72.9)	
**Conversion**	3/117 (2.6)	1/112 (0.9)		3/142 (2.1)	2/144 (1.4)	
**Onset to groin puncture time (min)**	277 (200–369) [*n* = 116]	242 (182–358) [*n* = 108]	−16.5	269 (197–334) [*n* = 142]	259 (201–332)	−1.9
**Onset to imaging time (min)**	150 (100–231) [*n* = 116]	136 (95–210) [*n* = 107]	−5.9	125 (97–167) [*n* = 141]	119 (95–152) [*n* = 140]	−14.2
**Imaging to groin puncture (min)**	130 (59–168)	93 (61–155) [*n* = 111]	−14.5	125 (77–184) [*n* = 143]	140 (78–193) [*n* = 141]	9

Abbreviations: ASPECTS = Alberta Stroke Program Early Computed Tomography Score; BP = blood pressure; ICA = internal carotid artery; SR = stent retriever.

Values expressed as no./total no. (%), mean (SD) or median (25th–75th percentiles).

**Figure 1:**
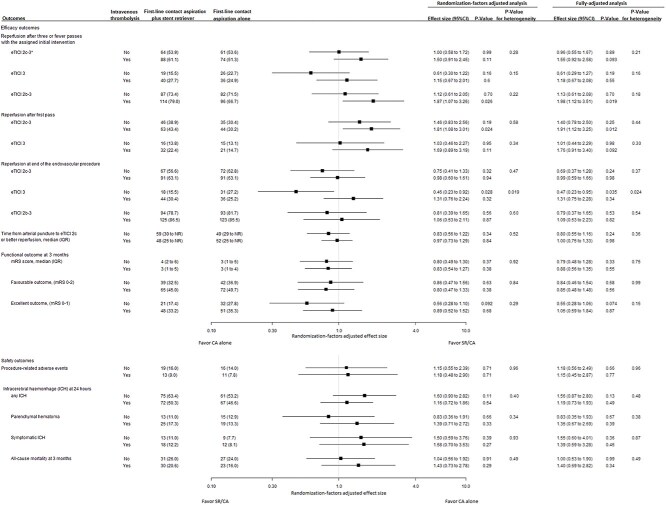
Treatment effect sizes on efficacy and safety outcomes for first-line stent retriever plus contact aspiration over first-line contact aspiration alone according to prior use or not of intravenous thrombolysis in patients with acute ischaemic anterior circulation stroke and positive susceptibility vessel sign. Descriptive values and effect sizes were calculated after handling missing outcomes values by multiple imputations. Effect sizes are expressed in term of adjusted odds ratio for binary outcomes, adjusted sub-hazard ratio for the time from arterial puncture to eTICI2c or better reperfusion and common odds ratio for 1-point improvement in mRS (after pooling together mRS 5 and 6). Randomisation factors adjusted effect sizes referred to effect sizes adjusted for randomisation stratification variables (centres, age (≤80 years vs >80 years), occlusion site (isolated MCA vs MCA and ICA) and use of general anaesthesia. Fully adjusted effect sizes referred to effect sizes adjusted for randomisation stratification variables and following additional baseline characteristics: current smoking, previous stroke/TIA history, antiplatelet therapy at inclusion, pre-stroke mRS and mode of admission (drip-and-ship vs mothership). Abbreviations: CA = contact aspiration, eTICI = expanded thrombolysis In cerebral infarction; ICA = internal carotid artery; NR = not reach, SR = stent retriever.

The effect of the first-line strategy (SR + CA vs CA alone) on the primary outcome (early near-complete or complete reperfusion [eTICI 2c-3] after 3 or fewer passes) did not differ significantly between IVT-treated and non-IVT-treated patients (*P* for heterogeneity = .28, Figure 1). In patients who received IVT, the primary outcome occurred in 61.1% in the SR + CA group vs 51.3% in the CA alone group (aOR = 1.50; 95% CI, 0.91–2.45). In patients who did not receive IVT, rates were similar between strategies (53.9% vs 53.6%; aOR = 1.00; 95% CI, 0.58–1.72). Similar findings were found after additional adjustment on current smoking, previous stroke or TIA history, pre-stroke mRS, antiplatelet therapy and mode admission or in secondary analysis before handling missing data by multiple imputation (Figure S1).

Across secondary angiographic endpoints, interactions tests were likewise non-significant for nearly all outcomes, indicating no evidence of heterogeneity in the first-line strategy according to the IVT status (Figure 1). This included reperfusion after 3 or fewer passes (eTICI 2b-3 and eTICI 3), reperfusion after first-pass (eTICI 2c-3 and eTICI 3) and time from arterial puncture to eTICI 2c or better reperfusion (all *P* for heterogeneity > .05). A significant interaction was observed for complete reperfusion (eTICI 3) at the end of MT (*P* for heterogeneity = .019). In patients without IVT, CA alone was associated with higher rates of complete reperfusion compared with the SR + CA strategy (27.2% vs 15.5%, aOR = 0.46; 95% CI, 0.23–0.92; *P* = .028), whereas no significant difference was observed among IVT-treated patients (30.4% vs 25.2%; aOR = 1.31; 95% CI, 0.76–2.24).

Although interaction tests were largely non-significant, treatment effects tended to favour the SR + CA strategy among IVT-treated patients. In this subgroup, numerically higher rates of successful reperfusion were observed with the SR + CA strategy for eTICI 2b-3 after 3 or fewer passes (79% vs 66.7% for SR + CA and CA alone, respectively, aOR = 1.87, 95% CI 1.07–3.26, *P* = .026), as well as for eTICI 2c-3 after first pass (43.4% vs 30.2%; aOR = 1.81; 95% CI, 1.08–3.01; *P* = .024). By contrast, among patients who did not receive IVT, treatment effects were generally neutral, with no consistent differences between first-line strategy across secondary angiographic endpoints.

There was no evidence of heterogeneity in treatment effect according to IVT status for any secondary clinical or safety endpoints (Figure 1).

Regarding the change in NIHSS at 24 h, the baseline-adjusted mean change was −5.0 (95% CI, −6.3 to −3.7) in the combined group vs −5.3 (95% CI, −6.5 to −4.0) in the aspiration-only group among IVT-treated patients, and − 2.9 (95% CI, −4.3 to −1.5) vs −3.8 (−5.2 to −2.3) among patients without IVT. In terms of first-line MT effect sizes, compared to contact aspiration, the combined strategy was not associated with greater NIHSS improvement whatever the IVT and no-IVT subgroups (Table 2).

**Table 2:** Effect of first-line stent retriever plus contact aspiration over first-line contact aspiration alone first-line thrombectomy strategy on 24-h NIHSS change according to prior use or not of intravenous thrombolysis.

IVT	SR + CA first-line strategy	CA alone first-line strategy	Adjusted mean between-group difference (95% CI)	*P* value	*P* value for heterogeneity
**Main analysis**					
**No**	−2.9 (−4.3 to −1.5)	−3.8 (−5.2 to −2.3)	0.9 (−1.1 to 2.9)	.36	.63
**Yes**	−5.0 (−6.3 to −3.7)	−5.3 (−6.5 to −4.0)	0.3 (−1.5 to 2.0)	.77	
**Complete case analysis**					
**No**	−2.8 (−4.2 to −1.3)	−3.8 (−5.3 to −2.4)	1.1 (−1.0 to 3.1)	.29	.61
**Yes**	−5.3 (−6.6 to −4.0)	−5.7 (−6.9 to −4.4)	0.4 (−1.4 to 2.2)	.66	
**Sensitivity analysis restricted to drip-and-ship patients**					
**No**	−1.9 (−3.8 to −1.7)	−2.3 (−4.3 to −0.3)	0.4 (−2.3 to 3.1)	.78	.94
**Yes**	−4.8 (−6.4 to −3.2)	−5.0 (−6.5 to −3.5)	0.2 (−2.5 to 2.9)	.86	

Abbreviations: CA = contact aspiration; ICA = internal carotid artery.

Values are adjusted mean change in NIHSS score from baseline to 24 h unless otherwise as indicated. Values and effect sizes (mean between-group difference) were adjusted for baseline NIHSS score and randomisation stratification variables: centres, age (≤80 years vs > 80 years), occlusion site (isolated MCA vs MCA and ICA) and use of general anaesthesia. Main analysis referred to multiple imputation analysis for handling missing outcomes values whereas sensitivity analysis referred to complete case available analysis.

An analysis restricted to patients admitted through a drip-and-ship paradigm is displayed in Figure S2 and did not show evidence of difference in effect sizes for combined SR + CA over CA alone between IVT and non-IVT-treated patients.

## Discussion

In this secondary analysis of the VECTOR trial, we found no statistically significant evidence that prior IVT modified the effect of the allocated first-line strategy (CA vs SR + CA) on near-complete or complete reperfusion (eTICI 2c-3) within 3 or fewer passes. Although the SR + CA strategy was associated with higher rates of early reperfusion in patients treated with IVT, interaction testing did not demonstrate a differential treatment effect according to IVT status. A statistically significant interaction between IVT status and treatment strategy was observed for complete reperfusion at the end of MT. Given the post hoc nature of these analyses and the absence of adjustment for multiple comparisons, the findings should be considered exploratory and hypothesis-generating. The observed heterogeneity in treatment effect size for final eTICI 3 may represent a chance finding, and we cannot exclude the possibility of a false-positive test for heterogeneity. Therefore, differences in treatment effect across subgroups should be interpreted with caution and require confirmation in independent studies. Of note, this interaction was not observed in the subgroup of patients admitted through a drip-and-ship paradigm (Figure S2).

However, a plausible, hypothesis-generating mechanism may nonetheless be proposed for this isolated finding. Because VECTOR enrolled only SVS-positive occlusions, a phenotype enriched for red blood cell-rich thrombi that are highly susceptible to fibrinolysis,^18^^,^^19^ the physical state of the clot at the time of MT may differ substantially according to prior IVT. In the absence of IVT, the clot retains its native cohesion: CA applied directly to an intact clot may favour *en-bloc* retrieval and limit fragmentation, whereas crossing and retrieving a firm clot with an added SR may promote fragmentation and distal or new-territory embolisation,^20^^,^^21^ thereby precluding truly complete reperfusion. Prior alteplase, by partially softening and degrading the clot, would attenuate this specific advantage of CA alone. This interpretation is concordant with the post hoc COMPASS analysis,^9^ in which prior alteplase was associated with lower final reperfusion in the aspiration-first arm but not in the stent-retriever arm. This mechanism remains speculative and warrants confirmation in dedicated prospective studies.

These findings address a clinically important and still unresolved question: whether treatment received before MT, particularly IVT, should influence the choice of first-line MT strategy. One of the main procedural concerns during MT is thrombus mobilisation, with the potential for clot migration, fragmentation and embolisation into a new territory. Intravenous thrombolysis may alter thrombus structure before catheter-based intervention, and thrombus migration or fragmentation after alteplase has been documented on serial vascular imaging.^20^^,^^21^ More broadly, stroke thrombi are heterogeneous, with variable proportions of red blood cell-rich and platelet/fibrin-rich components, and this composition influences both susceptibility to fibrinolysis and the mechanical interaction between the clot and MT devices.^18^^,^^19^ In theory, a first-line CA approach may reduce the need to cross the thrombus, thereby limiting mechanically induced clot displacement, whereas a combined approach, despite initial clot crossing with a SR, may provide stronger clot integration and a more controlled retrieval under simultaneous aspiration, potentially improving stability during extraction. This issue is clinically relevant, as optimising MT may require a more individualised approach that takes into account not only vascular anatomy and clot imaging features, but also reperfusion treatment administered before the procedure. Our findings should be interpreted in light of prior analyses that suggested a possible interaction between IVT and first-line MT technique, although the available evidence remain heterogeneous. In the post hoc analysis of MR CLEAN-NO IV trial by Rinkel et al.,^10^^,^^22^ IVT was the randomised treatment, whereas first-line MT technique was not. Among 473 patients who underwent MT, only 102 (21.6%) were treated with CA alone as first-line technique. The authors reported a significant interaction between IVT and first-line technique for 90-day functional outcome (*P* for interaction = .03): within the CA subgroup, patients treated with MT alone (ie, without IVT) had worse functional outcome than those treated with IVT + MT, whereas no such difference was observed in the SR group. By contrast, there was no statistically significant interaction for successful reperfusion. Importantly, MR CLEAN-NO IV only enrolled patients presenting directly to comprehensive stroke centres, which implies short needle-to-groin intervals and limits extrapolation to transfer settings. In our study, first-line technique was randomised, thereby reducing confounding related to operator-selected device strategy, and the cohort included both direct admissions and secondary transfers. This distinction may be clinically relevant, as longer transfer times may increase the duration of clot exposure to alteplase before MT and its effect on thrombus-device interactions.

In a COMPASS post hoc analysis including 235 patients, final TICI 2b-3 rates in the first-line CA arm were lower with prior alteplase than with MT alone (87.9% vs 100.0%; *P* = .03), whereas rates were similar in the first-line SR arm regardless of alteplase use (87.5% vs 87.5%).^9^ These observations are relevant but should be interpreted with caution. The analysis was post hoc and IVT exposure was not randomised. In addition, COMPASS compared first-line CA vs SR strategies, rather than CA vs a combined approach, which may better reflect current practice in many high-volume centres.^7^

From a practical standpoint, our data suggest that prior IVT use alone should not determine first-line MT strategy in anterior LVO. Factors driving rapid and effective reperfusion, such as operator experience, device availability and vascular anatomy, are likely more important factors. Consistent with this, we observed no clear differences in clinical and safety endpoints. Both CA- and SR + CA-based strategies therefore remain valid approaches, and in light of current evidence, IVT status alone should not guide first-line technique selection.

This study has some limitations. First, this analysis was retrospective. Consequently, subgroup differences and nominally significant findings for secondary angiographic endpoints should be regarded as exploratory, particularly given the multiplicity of comparisons. Second, IVT was not randomised in the VECTOR trial. Although the treatment strategy allocation was independent of IVT administration, IVT status was incorporated into the minimisation algorithm, thereby mitigating the risk of imbalance between first-line strategy groups. Third, the SR + CA strategy in VECTOR predominantly relied on a single stent retriever (EmboTrap), and SRs differ in radial force and clot-integration properties, so a device-specific effect cannot be fully excluded. However, head-to-head comparisons among the most widely used SRs have not shown significant differences in recanalisation or in embolisation to new territory,^23^ and EmboTrap met the efficacy benchmark derived from the pivotal Solitaire and Trevo trials.^24^ Our findings are therefore likely to extend to other commonly used devices. Fourth, both strategies were performed under standardised procedural conditions, including mandatory balloon guide catheter use. Such systematic proximal flow control may have reduced between-strategy differences and could limit extrapolation to settings where proximal flow control is not routinely applied. It is also worth noting that VECTOR enrolled only SVS-positive occlusions, a phenotype enriched for red blood cell-rich thrombi and previously associated with differential responses to MT techniques.^25^ These results should also be interpreted in light of the highly selected study population. VECTOR enrolled exclusively patients selected by MRI, and among them only those with a positive SVS; our findings therefore apply specifically to this MRI-selected, SVS-positive population and should not be extrapolated to patients selected by CT-based imaging or to SVS-negative occlusions. Because this phenotype was central to patient selection, it may have shaped both the response to IVT and the comparative efficacy of the 2 first-line strategies. Notably, VECTOR was conducted in France, where MRI is frequently used as the first-line imaging modality for acute stroke, which made systematic MRI-based selection feasible; in settings where CT predominates, such selection would be less readily applicable. Finally, IVT in VECTOR consisted predominantly of alteplase. Tenecteplase has since become an increasingly adopted^5^^,^^8^ and guideline-endorsed alternative in AIS with LVO.^14^^,^^26^ This caveat is more than theoretical: in a recent analysis of the AcT trial, the type of intravenous thrombolytic significantly modified the comparative efficacy of first-line CA vs SR, tenecteplase being associated with higher odds of final eTICI 2c-3 with aspiration but not with stent retriever.^27^ Consistent with a potentially distinct mechanism of action, tenecteplase has also been associated with superior early reperfusion compared with alteplase, most markedly in low-clot-burden lesions.^28^ The extent to which our findings, obtained almost exclusively under alteplase, apply to tenecteplase-based bridging therapy, particularly regarding device-clot interactions, therefore remains to be determined and warrants dedicated prospective evaluation.

## Conclusion

In conclusion, in this secondary analysis of the VECTOR trial, prior IVT use was not associated with a statistically significant interaction with first-line strategy for the prespecified primary angiographic endpoint. Overall, the relative efficacy of CA alone and SR + CA was largely consistent irrespective of IVT status, highlighting the need for dedicated prospective evaluation of how IVT may influence MT outcomes.

## Supplementary Material

Supplementary_material_aakag087

## Data Availability

The study protocol, statistical analysis plan, informed consent forms, and study data (including de-identified participant data and a data dictionary defining each field in the set) will be made available to othersupon formal request and receipt of a signed material transfer agreement.Requests should be directed to the corresponding author and will be assessed by the scientific committee of the VECTOR study. Data will be shared only via individual secured network connections.

## Notes

Clinical Trial Registration: NCT 04139486
