# Supplementary material for: Impact of prior intravenous thrombolysis on first-line thrombectomy strategy. A secondary analysis of the VECTOR trial
Source: Eur Stroke J. 2026 Jul 24;11(7):aakag087. doi: 10.1093/esj/aakag087 (PMC13398693; doi:10.1093/esj/aakag087)
Supplement: Supplementary_material_aakag087 [file supplementary_material_aakag087.zip › Supplemental Figure 1.docx]

**Supplemental Figure 1. Treatment effect sizes on efficacy and safety outcomes for first-line stent retriever plus contact aspiration over first line contact aspiration alone according to prior use or not of intravenous thrombolysis before handling missing outcome values by multiple imputations.**

Effect sizes are expressed in term of adjusted odds ratio for binary outcomes, adjusted subhazard ratio for the time from arterial puncture to eTICI2c or better reperfusion, and common odds ratio for 1-point improvement in mRs (after pooling together mRs 5 and 6). Effect sizes were adjusted for randomisation stratification variables (centers, age (≤80 years vs >80 years), occlusion site (isolated MCA vs MCA and ICA) and use of general anaesthesia.

Abbreviations: CA=contact aspiration, eTICI=expanded Thrombolysis In Cerebral Infarction; ICA=internal carotid artery; ICH=intracranial hemorrhage; IQR=interquartile range; MCA=middle cerebral artery; mRs=modified rankin scale; NR=not reach; SR=stent retriever.

**Supplemental Figure 2. Treatment effect sizes on efficacy and safety outcomes for first-line stent retriever plus contact aspiration over first line contact aspiration alone according to prior use of intravenous thrombolysis, in patients with acute ischaemic anterior circulation stroke, a positive susceptibility vessel sign and admission through a drip-and-ship paradigm.**

Descriptive values and effect sizes were calculated after handling missing outcomes values by multiple imputations. Effect sizes are expressed in term of adjusted odds ratio for binary outcomes, adjusted subhazard ratio for the time from arterial puncture to eTICI2c or better reperfusion, and common odds ratio for 1-point improvement in mRs (after pooling together mRs 5 and 6). Randomization-factors adjusted effect sizes referred to effect sizes adjusted for randomisation stratification variables (centers, age (≤80 years vs >80 years), occlusion site (isolated MCA vs MCA and ICA) and use of general anaesthesia. Fully-adjusted effect sizes referred to effect sizes adjusted for randomisation stratification variables and following additional baseline characteristics: current smoking, previous stroke/TIA history, and antiplatelet therapy at inclusion.

Abbreviations: CA=contact aspiration, eTICI, expanded Thrombolysis In Cerebral Infarction; ICA= internal carotid artery; ICH=intracerebral hemorhage; IQR, interquartile range; MCA, middle cerebral artery; mRs, modified rankin scale; NR, not reach, SR=stent retriever, TIA=transient ischemic attack.
