# Supplementary material for: Impact of prior intravenous thrombolysis on first-line thrombectomy strategy. A secondary analysis of the VECTOR trial
Source: Eur Stroke J. 2026 Jul 24;11(7):aakag087. doi: 10.1093/esj/aakag087 (PMC13398693; doi:10.1093/esj/aakag087)

**Supplemental Figure 1. Treatment effect sizes on efficacy and safety outcomes for first-line stent retriever plus contact aspiration over first line contact aspiration alone according to prior use or not of intravenous thrombolysis before handling missing outcome values by multiple imputations.**

Effect sizes are expressed in term of adjusted odds ratio for binary outcomes, adjusted subhazard ratio for the time from arterial puncture to eTICI2c or better reperfusion, and common odds ratio for 1-point improvement in mRs (after pooling together mRs 5 and 6). Effect sizes were adjusted for randomisation stratification variables (centers, age (≤80 years vs >80 years), occlusion site (isolated MCA vs MCA and ICA) and use of general anaesthesia.

Abbreviations: CA=contact aspiration, eTICI, expanded Thrombolysis In Cerebral Infarction; ICA, internal carotid artery; ICH, intracerebral hemorhage; IQR, interquartile range; MCA, middle cerebral artery; mRs, modified rankin scale; NR, not reach; SR=stent retriever.


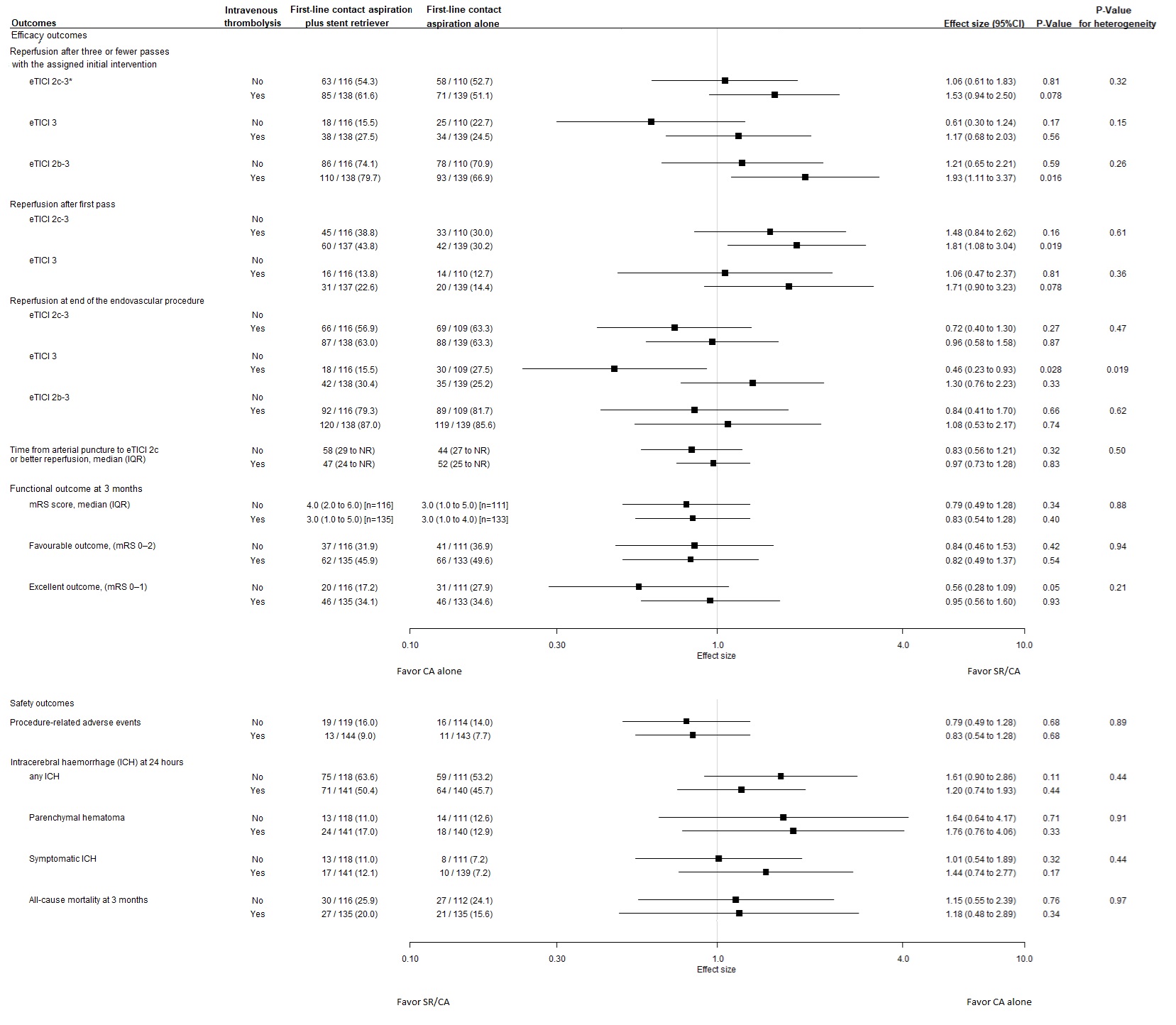

Supplement: Supplementary_material_aakag087 [file supplementary_material_aakag087.zip › Supplemental_Figure_1_aakag087.docx]
